# Integrating planar photonics for multi-beam generation and atomic clock packaging on chip

**DOI:** 10.1038/s41377-023-01081-x

**Published:** 2023-04-03

**Authors:** Chad Ropp, Wenqi Zhu, Alexander Yulaev, Daron Westly, Gregory Simelgor, Akash Rakholia, William Lunden, Dan Sheredy, Martin M. Boyd, Scott Papp, Amit Agrawal, Vladimir Aksyuk

**Affiliations:** 1 Physical Measurement Laboratory, National Institute of Standards and Technology, Gaithersburg, MD 20899 USA; 2 Department of Chemistry and Biochemistry, University of Maryland, College Park, MD 20742 USA; 3 Vector Atomic, Inc., Pleasanton, CA 94588 USA; 4 Physical Measurement Laboratory, National Institute of Standards and Technology, Boulder, CO 80305 USA

**Keywords:** Silicon photonics, Integrated optics, Atom optics

## Abstract

The commercialization of atomic technologies requires replacing laboratory-scale laser setups with compact and manufacturable optical platforms. Complex arrangements of free-space beams can be generated on chip through a combination of integrated photonics and metasurface optics. In this work, we combine these two technologies using flip-chip bonding and demonstrate an integrated optical architecture for realizing a compact strontium atomic clock. Our planar design includes twelve beams in two co-aligned magneto-optical traps. These beams are directed above the chip to intersect at a central location with diameters as large as 1 cm. Our design also includes two co-propagating beams at lattice and clock wavelengths. These beams emit collinearly and vertically to probe the center of the magneto-optical trap, where they will have diameters of ≈100 µm. With these devices we demonstrate that our integrated photonic platform is scalable to an arbitrary number of beams, each with different wavelengths, geometries, and polarizations.

## Abstract (web summary)

A planar platform combining photonic integrated circuits and flip-chip bonded meta-surfaces for multi-color light projection, beam shaping, and polarization control for compact laser cooling.

## Graphical abstract

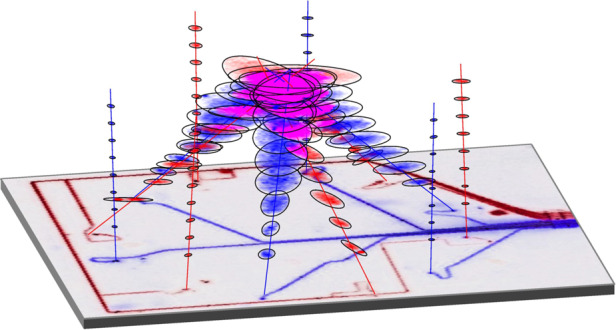

## Introduction

Optical systems form the backbone of atomic vapor, trapped ion, and neutral atom technologies. Precise control of the wavelength, power, and polarization of coherent free-space light is required for addressing the optical transitions in atomic systems. This control is readily achieved using laboratory-scale setups but becomes more challenging as optical systems are scaled down for commercialization. Miniaturized optical systems can be constructed using a combination of compact bulk^1^ or flat optics^2,3^, while photonic integrated circuits (PICs) may provide a scalable approach to manufacturing atomic technologies^4,5^.

PICs allow for foundry-scale integration of optical components that can range from laser sources and modulators all the way to on-chip detectors. Diffraction gratings can be integrated on chip to generate free-space beams from guided modes^6^ and used to address atomic systems out of plane^7^. Grating technologies have advanced over the years to enable multi-wavelength control of light^8–10^, surface-normal emission^11–14^, polarization control^15–17^, and the generation of beams with large numerical apertures^18,19^ and large mode expansions^20,21^. Yet challenges remain for combining these various capabilities within a single platform for power-efficient arbitrary beam control within a single integrated technology.

As an example, optical lattice clocks achieve state-of-the-art frequency instability and ultra-high timing accuracy^22–24^, but require a complex combination of bulk optics to generate the numerous laser beams and wavelengths needed to prepare the atomic sample used for the clock reference. A commonly employed scheme^25^ for lattice clocks based on strontium involves three physically overlapping optical cooling/trapping stages in sequence: a magneto-optical trap (MOT) at the wavelength of 461 nm for capture of thermal atoms, a smaller-volume MOT at 689 nm for further cooling, and an optical lattice trap at 813 nm for Doppler-free interrogation by a probe beam at the 698 nm clock transition^26,27^. Each MOT stage typically uses 3 pairs of counterpropagating beams to decrease the atomic momenta along all three spatial axes, with the lattice trap typically formed by a single beam and its retroreflection.

In this work, we design and fabricate an integrated photonics package for the miniaturization of a strontium atomic clock. Our approach is based on a bonded planar platform that combines optical metasurfaces (MSs) with grating outcouplers^28^ to produce beams with a high numerical aperture (NA), arbitrary tilt angles, prescribed polarizations, and collinear propagation. We demonstrate a compact photonic chip system generating twelve circularly polarized beams as large as 10 mm in diameter (461 nm). We design this system to form both the 461 nm and 689 nm MOTs within a small (25 mm)^3^ volume accommodating a vacuum chamber with strontium vapor. In addition, we demonstrate the collinear combination of two separately waveguided beams to produce an optical lattice (at 813 nm) aligned with a probe beam at the clock transition (at 698 nm). The combined lattice and clock beams are collinear to within 0.1° of one another and are vertically directed to within 1° from chip normal. Our planar platform provides a robust approach to beam generation on chip and represents a step towards realizing atomic technologies with manufacturable PICs. The demonstrated ability to simultaneously produce multiple carefully arranged beams with a variety of sizes, polarizations and wavelengths opens a path for scaling down the size and increasing complexity of atomic technologies beyond what is possible with discrete optical components.

## Results

### Atomic clock optical system design

**Fig. 1:**
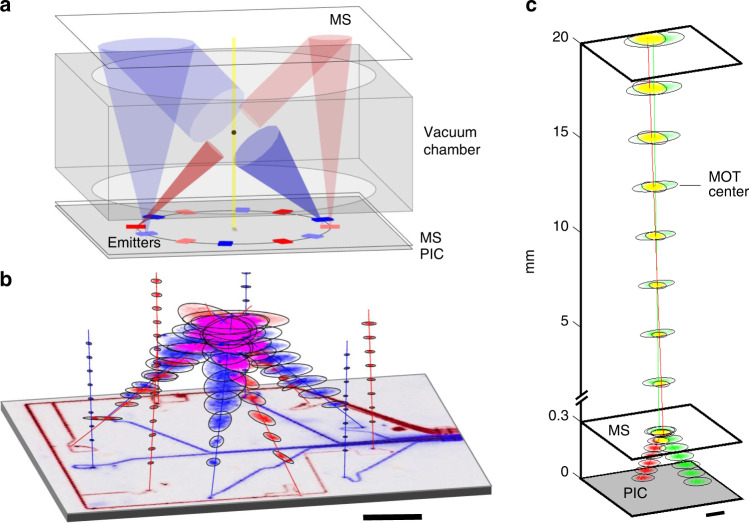
Sr atomic clock optical beams emitted by integrated photonics. **a** The photonics geometry as part of the strontium clock, showing the four types of beams including a pair of the red and blue beams at 0° (light color) and 45° (dark color), as well as the collinearly combined lattice and clock beams (yellow). **b** Measured trajectories of the twelve blue and red MOT beams, overlayed on an image of the PIC. Scale bar is 5 mm. **c** Measured trajectories of the lattice (green) and clock (red) beams below and above the MS (yellow region depicts the overlap of the two beams). Scale bar is 100 µm

We use two planar photonic chips to create all the beams necessary for three stages of laser cooling and trapping (two MOTs and one optical lattice) within an atomic clock. The blue MOT performs the initial laser cooling, followed by the red MOT, and finally by the vertically oriented optical lattice. The clock beam co-propagates with the optical lattice beam to measure the clock transition in the ultra-cooled Sr atoms. Our photonic chips are separated vertically by about 25 mm and located on top and bottom of a vacuum chamber with optical windows for introducing the beams. The MOT configuration for both colors (Fig. 1a) uses an unconventional arrangement of beams to obtain 3D cooling and trapping in a compact, planar geometry. A full diagram of all the MOT beams is provided in Fig. S1. The polarizations of the various beams are the same as in established 6-beam MOT configurations, while the beam diameters at the MOT location are about a factor of 2 smaller (details in Table 1). Adjustments are made to the propagation angles of the beams and the magnetic field axis is tilted off vertical to allow compact packaging. Magnetic field coils can be located immediately outside the photonic chips (or miniaturized coils placed within the chamber) and aligned with the left-hand circularly polarized counter-propagating beam pairs. Use of divergent MOT beams facilitates photonic integration. The MOT beams are generated to have the appropriate size at the position of the atoms while keeping the microfabricated emitters tractably small for improved efficiency. Blue and red emitter structures can be located in close proximity without constraints from the final beam size at the atoms, eliminating the need for complex dual-wavelength structures. Expanding beams are also well suited for 45° polar beam angles, which yield improved efficiency. Expanding-beam Sr MOTs have recently been demonstrated by the authors at both NIST and Vector Atomic^29^. Our planar photonic system produces wide, overlapping, counterpropagating beams with sufficient force along all axes for efficient atom cooling and capture.

**Table 1:** MOT and atomic clock system specifications

Specs	System	Blue MOT (461 nm)	Red MOT (689 nm)	Lattice (813 nm) / Clock (698 nm)
*Geometry*	• (25 mm)^3^ photonics package • Red & blue beams clocked by 35° • MOT coils to straddle the red and blue beam (LHC beams)	• 5 mm w_0_ • 3 emitters angled 45° from vertical • 3 emitters vertical	• 2 mm w_0_ • 3 emitters angled 45° from vertical • 3 emitters vertical	• ≈100 µm w_0_ • 1 vertical emitter each for lattice and clock
*Polarization*	• >75 % polarization fidelities	• 2 RHC, 1 LHC	• 2 RHC, 1 LHC	• Linear, lattice/clock cross polarized
*Power*	• <12 dB on-chip losses	• 3 mW per beam	• 1 mW per beam	• >100 mW (lattice) • ≈100 µW (clock)

The twelve bottom-up and top-down counterpropagating MOT beams can be emitted from a single PIC chip located at the bottom of the assembly. In this geometry, half of the beams are emitted up vertically to be redirected down at 45° by a top MS reflector as shown in Fig. 1a. The blue and red MOT beams are slightly azimuthally offset from one another (clocked by 35°) to spatially separate the beams to prevent overlap at the top reflector. Alternatively, two separate and identical PIC chips can be placed on the top and bottom of the vacuum chamber (see Fig. S2), replacing the top MS reflector but requiring two fiber-coupled photonic assemblies. We designed our photonic system to work with either configuration, emitting all 12 beams simultaneously, as seen in the experimental measurements in Fig. 1b.

Figure 1b shows all 12 beams generated to form the MOT device overlaid on a picture of the PIC chip with waveguides showing light propagation. Laser light is coupled into the chip from the right using a commercial V-groove fiber array with one fiber and waveguide per beam so the power of each beam can be tuned independently. Future designs will incorporate on-chip beam splitters to reduce the connectivity complexity and only require one input fiber per color. Figure 1c shows the measured lattice (green) and clock (red) beams combined to be collinear (yellow) using our compact emitter. The two beams are generated separately at the PIC surface and caused to overlap at the MS location. The MS acts as a birefringent wedge^30^ to tilt both beams to vertical, so they are collinear and overlap maximally at the MOT center.

### Device design

**Fig. 2:**
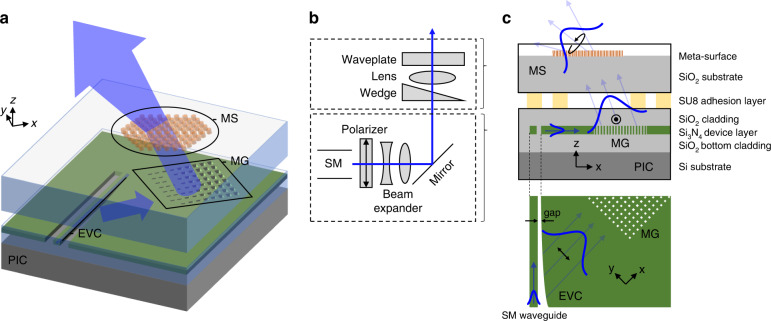
Integrated photonic emitters. **a** Three-stage design for the beam emitter consisting of an evanescent coupler (EVC), meta-grating (MG), and metasurface (MS) with each component contributing to beam shaping and polarization control. **b** The analogous free-space optics that the PIC and MS devices replace. **c** The EVC and MG are fabricated in a single silicon nitride device layer and function together as a polarizing beam splitter and a beam expander. Both top-down (bottom) and side (middle) views of the PIC devices are shown. The MS is fabricated on fused silica (top) and can be designed to function as a combination of an optical wedge, lens and waveplate. Polarization is denoted in black

Our beam emitters are constructed with a MS chip bonded onto a PIC chip (Fig. 2a). The PIC produces collimated beams directed off-normal from the chip surface with a pure linear polarization^20^. A patterned SU8 (see Commercial product disclaimer in Methods) photoresist layer is used as an adhesive to bond the PIC chip with a MS chip, which modifies the phase fronts, polarizations, and ultimate shape of the beams^31^. Multiple emitters are placed around a 25 mm diameter circle to produce the six blue and six red MOT beams. Half of the MOT beams are directed at ≈45° from the chip surface and intersect near the center of the volume at a height of ≈12.5 mm above the chip surface.

Each beam emitter consists of three components that perform separate functions (Fig. 2b). The first component is the evanescent coupler (EVC) that converts the waveguide mode into a collimated dielectric slab mode beam. This is achieved by adiabatically varying the gap between the on-chip single-mode (SM) waveguide (≈100 nm in width) and the dielectric slab until all the power is converted into the slab^20^ (Fig. 2c, bottom panel). By varying the gap separation between the waveguide and the slab mode, we shape the outgoing slab mode beam intensity to have a Gaussian profile (along the y-direction). The slab mode beam is offset from the waveguide direction by 31° (24°) for the blue (red) wavelength and TE polarization devices. This offset also separates the TE from the TM polarized modes into different slab-mode angles such that only the desired TE polarization is incident normal on the second component, the meta-grating (MG). Our MG is designed with subwavelength elements to provide fine control of the outcoupling strength^32^. By apodizing the size of the MG elements, the profile of the free-space beam is shaped along the x-direction. Since beam shaping is separated into these two orthogonal directions (x and y), arbitrary beam profiles can be created. Both the EVC and the MG are fully etched from a single silicon nitride layer and clad with oxide on the top and bottom (Fig. 2c). Together, the EVC and MG function as a SM beam expander with an integrated polarizing beam splitter. We design the EVC and MG to emit a nominally collimated beam into free space. This is accomplished by using a straight EVC with a uniform waveguide cross-section to produce a collimated slab-mode beam, and then by carefully apodizing the MG period to maintain collimated emission into free space^20^. The third component of the beam emitter is the MS optic, which is fabricated on a fused silica chip and bonded on top of the PIC using a patterned polymer adhesion layer. The MS is defined with TiO_2_ nanopillars whose lateral dimensions we design to function as an optical wedge to tilt the beam, a lens to focus or defocus the beam, and a waveplate to change the polarization of the beam. Details of the PIC and MS fabrication as well as the flip chip bonding are included in the Materials and Methods section.

The EVC, MG, and MS structures complete our compact beam emitter, which provides robust control of the generated free-space beam profile: its divergence, tilt angle, and polarization. By combining the large mode expansion of the EVC and MG elements^20,32^ with the large NA of the MS element^33^ we expand a micron-scale waveguide mode into a centimeter-scale free-space beam at a height of ≈12.5 mm above the chip surface.

### Device performance

Measurements of both test devices and the MOT chips provide a breakdown of loss in our system. The fiber couplers incur ≈5 dB (≈3 dB) loss while the propagation losses are measured to be ≈2 dB/cm (≈0.5 dB/cm) at the 461 nm (689 nm) wavelength. The grating outcouplers introduce ≈3 dB loss for both colors. Separate free-space measurements of the MS devices indicate that they introduce ≈2 dB to 3 dB excess transmission loss. Therefore, the longest beam path in the blue (red) experience cumulative loss of ≈15 dB (10 dB) from fiber to MOT center. Multiple flip chip bonding tests indicate that we achieve 7 µm ± 3 µm translational misalignment and 3 µm ± 2 µm rotational misalignment, root-mean-square (RMS), between the PIC and MS chips. These are measured with alignment marks near the 25 mm diameter circle that defines our MOT geometry. The quoted uncertainties are one standard deviation statistical uncertainty estimates based on repeat experiments.

**Fig. 3:**
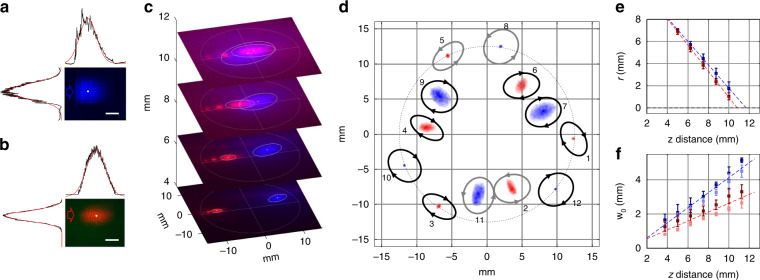
Measured MOT beam performance. **a** Image of a blue 45° beam, showing integrated power profiles projected along the horizontal and vertical directions along with Gaussian fits (red curves). **b** Image of a red 45° beam, showing beam profiles and Gaussian fits (red curves). Scale bar for all panels is 100 µm. Arrows depict the direction of the slab-mode beam incident on the grating. **c** Images of one red and one blue 45° beam taken at different heights above the chip. The red light has three diffraction orders visible, positive (left), zero (center), and negative (right, circled). **d** Map of the twelve beams measured at ≈4 mm above the MS. Overlayed with each beam is the optical field polarization measured normal to the beam propagation direction and plotted here as ellipses in the chip plane with right-handed circular polarization (black) and left-handed circular polarization (gray) indicated. In all (**a**–**d**) the image intensities have been rescaled from unsaturated images for best viewing of the beam shapes and trajectories. A complete data set of unsaturated images are provided in Fig. S3. **e** Average radial position of the red and blue 45° beams at different heights. Error bars correspond to the standard deviation of the three measured beams. **f** Measured cross-sectional w_0_ for the red and blue 45° beams with light and dark colors corresponding to w_0_ along the azimuthal and radial directions, respectively. Error bars correspond to the standard deviation of the three measured beams

A summary of the blue and red MOT beam performances is shown in Fig. 3. The blue beams are designed to emit at either 33.7° or 10.3° from the PIC surface normal depending on whether they will be redirected to 45° or 0° by the MS, respectively. We measure these angles to be 32.3° ± 0.8° (10.5° ± 0.5°) for the fabricated blue 45° (0°) beam devices. Similarly, the red beams are designed to emit at 30° (8°) and are measured to be 31.0° ± 0.3° (7.1° ± 0.7°). Unless noted otherwise, quoted uncertainties are one standard deviation statistical uncertainty estimates based on measurements of the three identical devices on the MOT chip. The beams are designed to have an elliptical Gaussian beam profile with a 1/e^2^ radius of the beam intensity, defined as w_0_, of 141 µm (100 µm) along the x (y) directions. The elliptical shape is chosen for all beams because it produces a symmetric cross section with w_0_ = 100 µm as viewed at 45°. Figure 3a shows an image of a blue 45° beam emitted from the PIC surface. The w_0_ is measured to be 129 µm ± 7 µm (100 µm ± 5 µm) along the x (y) directions. The blue beam shape is slightly asymmetric due to pronounced scatter at the front of the grating, where the fabrication precision limits further reduction of the outcoupling strength^32^. A red 45° beam is shown in Fig. 3b with w_0_ calculated to be 140 µm ± 4 µm (83 µm ± 7 µm) along the x (y) directions. Not shown are the profiles of the 0° beams for blue and red, which have w_0_ of 65 µm ± 6 µm (102 µm ± 3 µm) and 118 µm ± 9 µm (77 µm ± 8 µm), respectively, along the x (y) directions. Current chip designs prioritize the performance of the blue 45° beams.

Characterization of the beam performances after bonding the MS chip are shown in Fig. 3c–f. Figure 3c shows the raw images of a pair of blue and red 45° beams at different heights above the MS surface. A two-dimensional Gaussian profile is fitted to the main diverging beams, which is shown as an ellipse (white) whose major and minor axis radii are equal to w_0_ as fitted along the radial and azimuthal directions, respectively. Various diffraction orders of the MS can also be seen in Fig. 3c, with the zero-order and negative-order modes for the red 45° beam being most pronounced. The zero-order mode propagates along the trajectory of the original PIC beam, at the nominal 30° angle. The MSs are designed to strongly suppress these unwanted orders, but they can arise due to process imperfections. To preserve systems performance, our layout is designed to ensure that these unwanted modes avoid the MOT region and do not affect laser cooling performance. These unwanted orders can be a source of power loss in our system with the measured relative power lost into the higher orders of 0.4 % ± 0.3 % for the blue beams and 45 % ± 20 % for the red beams. Although both blue and red MSs are designed for optimal performance, the red MSs exhibited reduced performance and larger variability within the fabricated device. Variability in emitter-to-emitter power performance can be compensated by adjusting the power at the fiber input to the chip. MS performance over a broader wavelength range could be improved with changes to the meta-element thickness, which we plan for future devices.

Figure 3d is an image of the twelve beams at ≈4 mm above the MS chip surface (Fig. S3 shows the beams at additional heights). These beams are numbered according to the waveguide that leads to them (from top to bottom at the edge of the chip). Beams 8, 10, and 12 (1, 3, and 5) correspond to the 0° blue (red) beams and are measured with a polar angle of 1.6° ± 0.1° (2.2° ± 0.1°) and 70 % ± 10 % (33 % ± 3 %) degree of circular polarization (DOCP). The DOCP is the percentage of the beam power that has the correct circular polarization. Beams 7, 9, and 11 (2, 4, and 6) correspond to the blue (red) beams that are designed to emit at 45° and are measured with a polar angle of 46.6° ± 0.3° (49.7° ± 0.4°) and a DOCP of 66 % ± 6 % (43 % ± 16 %). Due to the symmetry breaking required for MOT laser cooling, beams 2, 5, 8, and 11 are left-handed circular polarized while all the rest are right-handed circular. Figure 3e plots the radial position of all the 45° beams as a function of height above the MS. The blue (red) beams are measured to intersect at a height of 11.68 mm ± 0.04 mm (10.80 mm ± 0.06 mm) above the MS chip, compared to the desired 12.5 mm. At this intersection height, the beams have an average w_0_ of 4.9 mm ± 0.1 mm (2.9 mm ± 0.1 mm) for the blue (red) beams, compared to their ideal sizes of 5 (2) mm. Figure 3f shows the cross-sectional w_0_ of the beams along the radial and azimuthal directions. The cross-sectional w_0_ is calculated by fitting the image to a two-dimensional Gaussian profile. The radial cross-sectional w_0_ is scaled by 1 / $\sqrt 2$ to compensate for the 45° tilt of the beam, while the azimuthal cross section is unscaled. Both the red and blue beams are slightly elongated along the radial direction. This asymmetry can be compensated by reducing the beam w_0_ along the grating direction in future PIC devices.

**Fig. 4:**
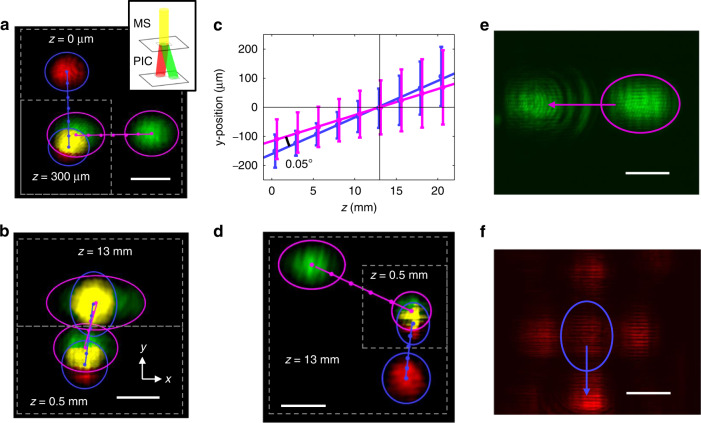
Lattice and clock beam performance. **a** Composite image of the lattice (green) and clock (red) beams taken at the PIC surface (z = 0 µm, large white box) and the intersection height (z = 300 µm, small white box). The inset illustrates the ideal performance of the beam combiner. **b** Composite image of the lattice and clock beams measured at the MS surface (z = 0.5 mm, bottom white box) and near the MOT center (z = 13 mm, top white box). **c** Measured y-position of the lattice (magenta) and clock (blue) beams as a function of height above the MS. The vertical bars of the data points correspond to the average w_0_ of the beams. **d** Composite image of the lattice and clock beams (similar to **b**) for a MS that is mismatched by 1° in wedge correction. **e** Image of the desired negative-order lattice beam (circled) and zero-order beam (at arrow) taken 350 µm above the MS. **f** Image of the desired negative-order clock beam (circled), zero-order beam (at arrow), and other order beams taken at 200 µm above the MS. All images are false colored, and all scale bars are 100 µm

The performance of the lattice and clock beam combiner is summarized in Fig. 4. This beam combiner also consists of a PIC chip and a MS chip but are fabricated separately from the previously described MOT chips. We use a mechanical stage to shift the MS chip and test different MS designs with the same PIC device. The PIC device consists of an emitter at 813 nm (green) and another at 698 nm (red), both designed to produce collimating beams angled at 22° from chip normal. The two emitters are oriented 90° relative to one another so that the beams have orthogonal polarizations when they overlap. When the MS is placed above the PIC, the beams overlap at the height of the MS, which acts as a polarization-multiplexed birefringent wedge that tilts both beams vertical. Figure 4a shows the beam trajectories before the MS chip is placed. The beams overlap at a height of ≈300 µm in free space above the PIC, which is the expected height given the difference in the refraction index between air and the ≈500 µm thick fused silica MS substrate. Figure 4b shows the trajectories of the beams after the MS. The two beams have a separation angle of ≈0.05° and propagate together at an angle of ≈0.6° from chip normal. While the beams are slightly misaligned at the MS plane (z = 0.5 mm), the slight separation angle brings them into near-perfect overlap at the nominal MOT position (z = 13 mm). Figure 4c plots the average w_0_ and trajectories of the beams along the y-direction, the direction of ≈0.6° tilt, showing that the two beams are well overlapped throughout the region where the red MOT beams overlap. Atoms are loaded into the optical lattice trap from the red MOT. The lattice and clock beams are offset by only ≈100 µm from the red MOT geometric center, which can be easily compensated with bias field coils and should not impact transfer efficiency into the lattice. The small misalignment between the lattice and clock beams is not expected to significantly affect atomic clock performance. Figure 4d plots the trajectory of the beams when an improperly matching MS, with wedge angles off by ≈1° for both the lattice and clock beams, is placed above the PIC. The resulting beams are no longer collinear and diverge far from each other at the nominal MOT location.

Images of the diffraction orders produced by the MS are shown in Fig. 4e, f for the 813 nm lattice and 698 nm clock beams, respectively. For the lattice beam, the MS produces only the desired negative-order beam and the undesired zero-order beam, which correspond to ≈52 % and ≈38 % of the upward measured power, respectively. The clock beam, however, is split into several higher order beams with ≈12 % and ≈26 % of the power going to the desired negative-order beam and the zero-order beam, respectively. The current performance is consistent with our strontium atomic clock requirement that the lattice beam be more power efficient than the clock beam. The observed undesirable modes can be suppressed by optimizing the MS element shape, inter-element spacing, and choice of the MS element thickness. As with the zero order MOT beams, the zero and higher order lattice and clock beams diffract at angles that do not interact with atoms within the MOT.

### Misalignment analysis

**Fig. 5:**
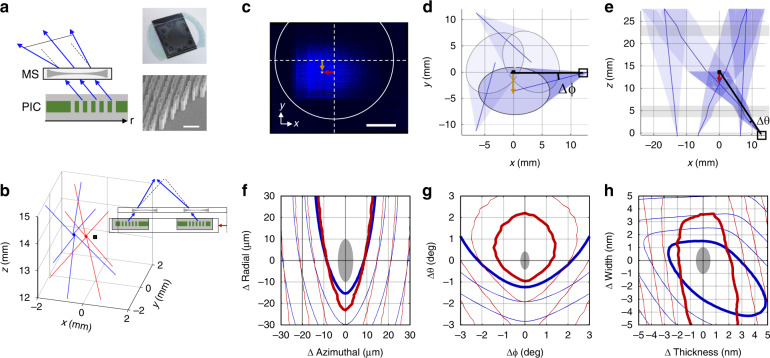
Misalignment analysis of the MOT beams. **a** Diagram of PIC and MS alignment. The insets show a picture of the bonded device (top) and an SEM image of MS nanopillars (bottom, scale bar is 500 nm). **b** Simulation of beam misalignment due to a translational offset of 10 µm. Lines correspond to the axes of the six beams for the 461 nm (blue) and 689 nm (red) MOTs. The black point marks the MOT center for a perfectly aligned system. **c** Image of a blue beam at the height of the MS with an overlay depicting misalignment to the MS (centered at white crosshairs). The red arrow indicates a -40 µm radial misalignment, while the yellow arrow indicates a clockwise 40 µm azimuthal misalignment. Scale bar is 100 µm. **d** Simulated top view of the three blue 45° beams with a 40 µm clockwise azimuthal misalignment for illustration. The yellow arrow highlights the beam shift at the MOT center. **e** Simulated side view of all blue beams with a -40 µm radial misalignment. The red arrow highlights the beam shift at the MOT center. Gray regions depict the glass windows for a simulated vacuum chamber. Black squares highlight the location of the beam whose example offset is shown in (**c**). **f** Normalized beam overlap volume as a function of radial and azimuthal position misalignment between the PIC and MS emitter elements for the 461 nm (blue) and 689 nm (red) MOTs. **g** Normalized beam overlap volume as a function of angular misalignment. **h** Normalized beam overlap volume as a function of device fabrication imprecision. Contour spacings are 20 % with the 80 % contour bolded in (**f**–**h**). Gray regions in (**f**–**g**) correspond to typical experimental misalignments and the corresponding estimated fabrication imprecision (**h**)

Misalignments between the PIC emitters and the MS elements can cause large deviations in the beam trajectories. The lattice and clock combiner is insensitive to translation errors, while rotational misalignment only cause proportional deviations to the beam trajectory (e.g., a 1° misalignment only causes a 1° change in the beam trajectory). In contrast, misalignments between the PIC and MS elements in the MOT emitters can cause compounding misalignment, with spatial misalignments also causing angular misalignments and vice versa. This is because the MSs in the MOT emitters act as very strong defocusing lenses, which expand the beams to the centimeter-scale sizes required for laser cooling (Fig. 5a). For the blue MOT emitters, a lateral misalignment of 10 µm between the PIC and MS elements leads to a ≈0.84 mm shift in the beam center position at the MOT. A similar misalignment for the red MOT emitters leads to only a ≈0.37 mm shift, which is less sensitive due to the weaker beam expansion required at this wavelength. Vertical misalignments between PIC and MS elements are less problematic due to both the steep beam angles, which do not cause large lateral beam shifts, and the fine control we have of the adhesive thickness (Fig. S4).

There are several causes of misalignment that affect the performance of the MOT emitters. First is the inaccuracy of the flip-chip bonding procedure and second are the inaccuracies of the EVC and MG operation. The inaccuracies of the MS operation are typically negligible compared to the PIC devices because the performance of the MS phase modulation is robust against fabrication imprecisions, which typically only result in reduced polarization control, higher MS reflectivity, and more power lost to higher order diffraction modes. In contrast, both spatial and angular operation of the EVC and MG devices are sensitive to fabrication imprecision.

Due to the symmetric arrangement of the MOT beams on our chip, there are three forms of systematic misalignments that can arise in our system: translational misalignment, azimuthal (i.e., rotational) misalignment, and radial misalignment. Inaccuracy in flip-chip bonding produces both translational and azimuthal misalignments, while fabrication imprecision on the PIC produces azimuthal and radial misalignments. Translational misalignment results from a systematic displacement between the PIC chip and the MS chip during flip chip bonding and causes all the MS lenses to be shifted from the PIC emitters by the same amount in the same direction. All beams of the same color are shifted similarly and intersect one another at the same location, albeit at an offset position from the desired MOT center. Figure 5b shows a diagram of how translational misalignment in the x-direction can cause the centers of all the blue beams and all the red beams to intersect at offset positions. Current flip chip bonding procedures result in ≈7 µm RMS translational misalignments, which corresponds to less than 1 mm offsets of the MOT center. These millimeter-scale offsets can be compensated by fine tuning the location of the magnetic fields in the final integrated clock system and should not significantly affect MOT performance.

Systematic azimuthal and radial misalignments, however, cannot easily be corrected. Therefore, it is important to estimate their effects on the MOT performance. Our current flip chip bonding procedure produces azimuthal misalignments with ≈3 µm RMS error at the emitter locations, which result from a systematic rotation between the MS and PIC chips when bonding. Azimuthal and radial misalignments also occur due to the imperfect fabrication of the EVC and MG devices, respectively. Examples of these two types of systematic misalignments are shown in Fig. 5c–e. If imperfect fabrication causes all the EVCs to emit slab mode beams offset in the negative y-direction (relative to the MS optic), then global clockwise azimuthal misalignments like those shown in Fig. 5d occur. Here, all the beams move out from the center of the MOT, progressively missing one another with larger misalignment. In contrast, if imperfect fabrication causes all the MGs to emit beams early (in the negative x-direction) then global radial misalignments like those shown in Fig. 5e occur. Here, the beams intersect at a lower z-height above the chip, causing a smaller beam overlap volume. Experimentally measured variations in identical EVC and MG designs across identically fabricated chips indicate a typical azimuthal and radial misalignment of ≈3 µm and 11 µm RMS, respectively.

The effects of azimuthal and radial misalignments on the overlap volume of the MOT beams are shown in Fig. 5f. These data are obtained through ray tracing simulations of the misaligned MOT geometry. Contours for both the blue and red MOTs are overlaid. Current calculations of our azimuthal and radial misalignments are depicted by the gray ellipse, which estimates no more than a 20 % loss in overlap volume for our typical fabricated devices. Our analysis assumes that the overlap volume is a good figure of merit for estimating MOT performance. Atom capture number typically scales with the size of the beams to a power of 3.6^34^. The present analysis, limited to a discussion of the overlap volume, does not account for the tradeoff between the MOT volume and the finite beam intensity, which becomes complicated for divergent beams^1^. This assumption can give misleading results when our geometry has positive radial misalignments (see top half of Fig. 5f). At some positive radial misalignment value, the finite intensity of the beams will reduce atom capture even as the overlap volume continues to increase. A more accurate study of misalignment effects would require atom capture simulation, which is beyond the scope of the current work.

Similar analysis is performed for angular misalignments along the azimuthal and radial directions due to imprecision in the EVC and MG performance, respectively. Figure 5g plots the contours of the normalized overlap volume and compares it to our experimentally measured angular misalignments of ≈0.2° (≈0.4°) RMS for the azimuthal (radial) angles, φ (θ), as shown in gray. Due to the free-space propagation of the beams in the separation distance between the PIC and MS chips, angular misalignments of the EVC and MG cause both a spatial and angular misalignment as the beams propagate to the MOT center.

Figure 5h combines the ray tracing model with finite element electromagnetic models of the EVC and MG optical designs to calculate the effects of fabrication imprecision on overlap volume. We consider systematic variations to the device-layer thickness as well as variations to the width of etched device-layer elements and calculate the affects to the spatial and angular performance of both the EVC and MG devices. This analysis accounts for the interplay between spatial and angular misalignments that can be compounding or counteracting depending on systematic deviations of the fabricated geometry from nominal design, which is common to both EVC and MG devices since they are fabricated with the same lithography step. This analysis provides an estimate for the required fabrication precision and an estimate for how well our current fabrication performs based on the experimentally measured misalignments (previously shown in gray in Fig. 5f–g). Changes of <20 % to the MOT overlap volume typically require better than 1 nm to 2 nm fabrication precision, although a bias in the design towards a thicker device layer and slightly thinner etched element sizes might relax this requirement to ≈4 nm precision.

## Discussion

In this paper, we demonstrated a scalable approach to fabricating the photonics for a miniature strontium atomic clock. Our approach is based on a planar architecture that combines the manufacturability of PICs with the versatility of MSs bonded on top to produce optical systems with multiple beam emitters on chip for a high degree of control over the polarization, tilt, and divergence of the emitted free-space beams. This integrated design offers a scalable approach to realize increasingly complex optical systems with many beams of multiple colors and functions without additional fabrication steps. We also demonstrated a novel co-linear beam combiner based on this planar technology and illustrated how each element provides unique capabilities to the fabrication of miniaturized atomic clocks. We performed a comprehensive analysis of our system performance as it relates to element misalignment and fabrication imprecision and found the measured misalignments to fall well within the range required for acceptable MOT performance.

Future work is focused on integrating the photonics package with a vacuum chamber for physics experiments as well as in making PIC designs that are compatible with foundry-scale lithography. Focus will also be placed on improving photonic losses at 461 nm to ensure commercial appeal compared with laboratory-scale setups. The current MOT PIC is large, with the longest blue waveguide being about 35 mm. Being able to make a large PIC is advantageous for creating complex arrangements of large free space beams, however waveguide losses, particularly at short wavelengths, become a major challenge. The 461 nm MOT requires at least 3 mW of power in each beam and linear and nonlinear losses at this wavelength in our Si_3_N_4_ material have prevented us from reaching sufficient emitted power in the beam with the longest waveguide and demonstrating atom capture with the present device. Importantly, this is not a fundamental limitation of the relatively compact emitter technologies, but a technical limitation of long waveguides. It can be overcome in the future devices by adding dedicated waveguiding layers using materials with low loss in the blue or thin Si_3_N_4_ waveguides pushing the mode energy out into the low-loss oxide cladding^35^. Some of our initial tests demonstrate exceptionally reduced propagation losses with 30 nm thick Si_3_N_4_ waveguides (see Fig. S5). In addition to propagation losses, polarization impurity is also limiting our potential MOT performance. Yet, several of our recently improved MS designs can convert linear to circular polarization with 90 % ± 4 % (94 % ± 2 %) DOCP for the blue (red) beams, in devices not yet integrated with PICs.

The demonstrated planar assembly of PICs with bonded MSs provides a unique platform for fabricating scalable technologies that extend beyond atomic clock designs. Grating couplers on PICs can be integrated not only with MSs, but also with on-chip modulators^36^ and lasers^37^ and patterned into large arrays for fabricating devices that perform complex dynamic control of optical fields in free space^38^. Many of the desirable features of free-space beam control, such as surface-normal emission and arbitrary polarization control, are difficult to achieve with simple fabricated grating outcouplers. However, by apportioning part of this control to an added MS element the system design space is opened, enabling devices that are more power efficient and manufacturable.

## Materials and methods

### PIC fabrication

PIC devices are fabricated on an oxidized silicon wafer. The oxide is grown to a nominal thickness of 2.7 µm using a wet thermal oxidation process. The device layer consists of stoichiometric Si_3_N_4_ grown using low-pressure chemical vapor deposition (LPCVD) to a nominal thickness of 100 nm. The EVC and MG elements as well as the fiber couplers at the facet of the chip are patterned using electron beam lithography (EBL) while the waveguides are patterned using a subsequent photolithography step. The fiber couplers include subwavelength structures to expand the waveguide mode to better match that of the fiber^39^. All structures are etched using a single reactive ion etch step through the entire nitride layer thickness. The device layer is then overgrown with a low temperature oxide cladding to a nominal 2.5 µm thickness using LPCVD. The wafer is then diced, and the edges polished to expose the fiber couplers. We use a 12-channel angle-polished v-groove fiber array to couple to all blue and red waveguides simultaneously.

### MS fabrication

The MS optics feature high aspect-ratio TiO_2_ nanopillars that are fabricated using EBL followed by a Damascene process based on atomic layer deposition^40,41^. The lattice-clock-combining MS optic consists of high aspect-ratio Si nanopillars that are fabricated using EBL and inductively coupled plasma reactive ion etching^5^.

### Flip chip bonding

An SU8 layer is used as an adhesive for flip-chip bonding. It is spun to a nominal 9 µm thickness and patterned to leave 30 µm diameter pillars arrayed with 20 µm separations. Windows in the SU8 around the gratings are left unpatterned to ensure that light does not pass through the resist. We use a commercial flip chip bonder to align and bond the MS chip to the PIC chip. The PIC chip is held at 125 °C and then raised to 150 °C when brought in contact with the MS chip^42^, which is then pressed into the PIC using ≈980 N of force (nominal 10 kg weight equivalent) for 1 min.

### Disclaimer

All references to commercial products in this paper are provided only to document how results have been obtained. Their identification does not imply recommendation or endorsement by NIST.

## Supplementary information

Supplementary information file

## Data Availability

Data in this manuscript is available at (10.18434/mds2-2922).
