# Supplementary material for: Integrating planar photonics for multi-beam generation and atomic clock packaging on chip
Source: Light Sci Appl. 2023 Apr 3;12:83. doi: 10.1038/s41377-023-01081-x (PMC10068800; doi:10.1038/s41377-023-01081-x)
Supplement: Supplementary file 1 — Supplementary information file [file 41377_2023_1081_MOESM1_ESM.docx]

Supplementary Information for

Integrating Planar Photonics for Multi-Beam Generation and Atomic Clock Packaging on Chip

Chad Ropp^1,2^, Wenqi Zhu^1^, Alexander Yulaev^1,2^, Daron Westly^1^, Gregory Simelgor^1^, Akash Rakholia^3^, William Lunden^3^, Dan Sheredy^3^, Martin M. Boyd^3^, Scott Papp^4^, Amit Agrawal^1^, and Vladimir Aksyuk^1*^

^1^Physical Measurement Laboratory, National Institute of Standards and Technology, Gaithersburg, Maryland 20899, USA

^2^Department of Chemistry and Biochemistry, University of Maryland, College Park, MD 20742, USA

^3^Vector Atomic, Inc., Pleasanton, California 94588, USA

^4^Physical Measurement Laboratory, National Institute of Standards and Technology, Boulder, Colorado 80305, USA

Included data: 5 Supplementary figures

**
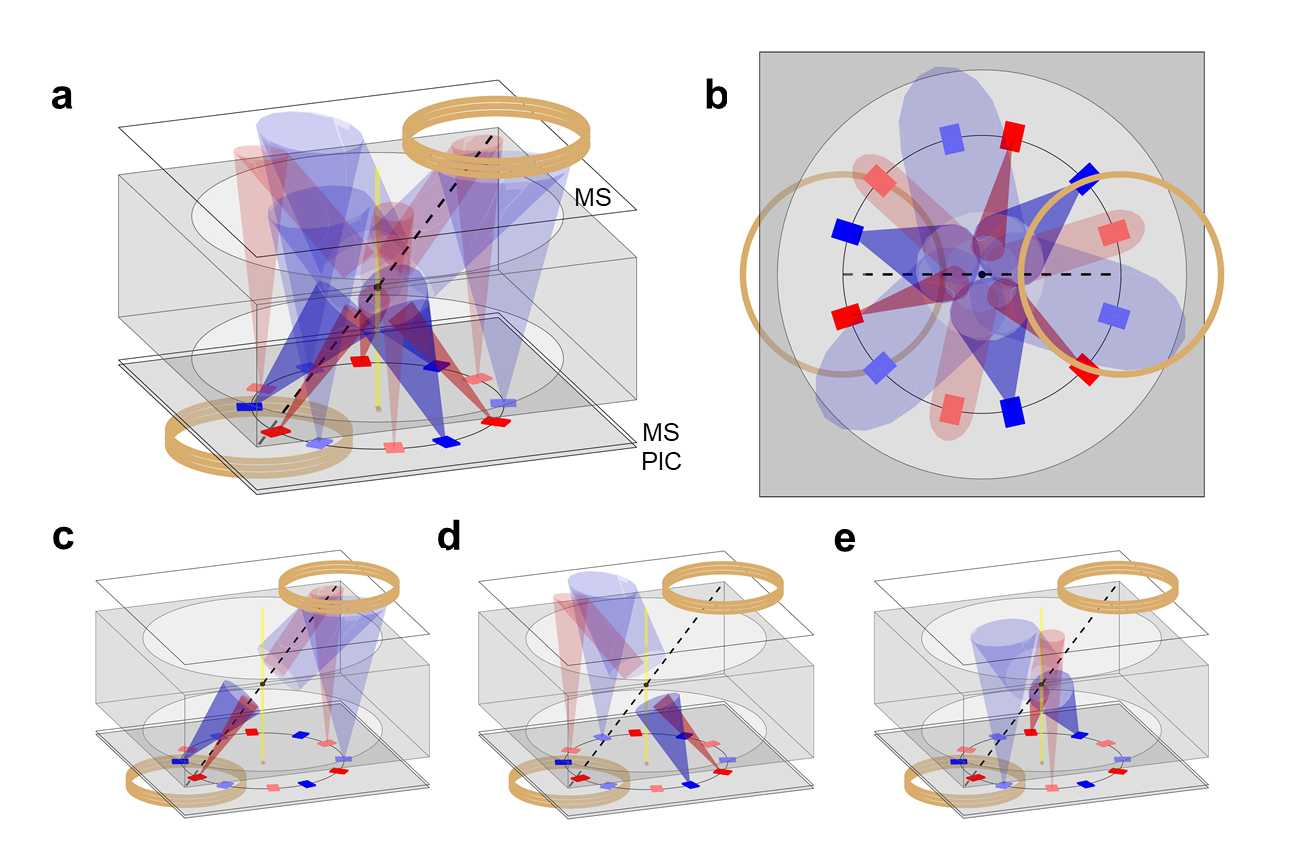
**

**Figure S1. Single-sided PIC design**. **a** Perspective view of the blue and red MOT beams along with the lattice and clock beams (yellow). The black line denotes the orientation of the magnetic field coils, which is aligned to the midpoint of the left-hand circularly polarized beam pairs. **b** Top perspective view of the design. **c-e** Perspective view of the design with different beam pairs shown.

**
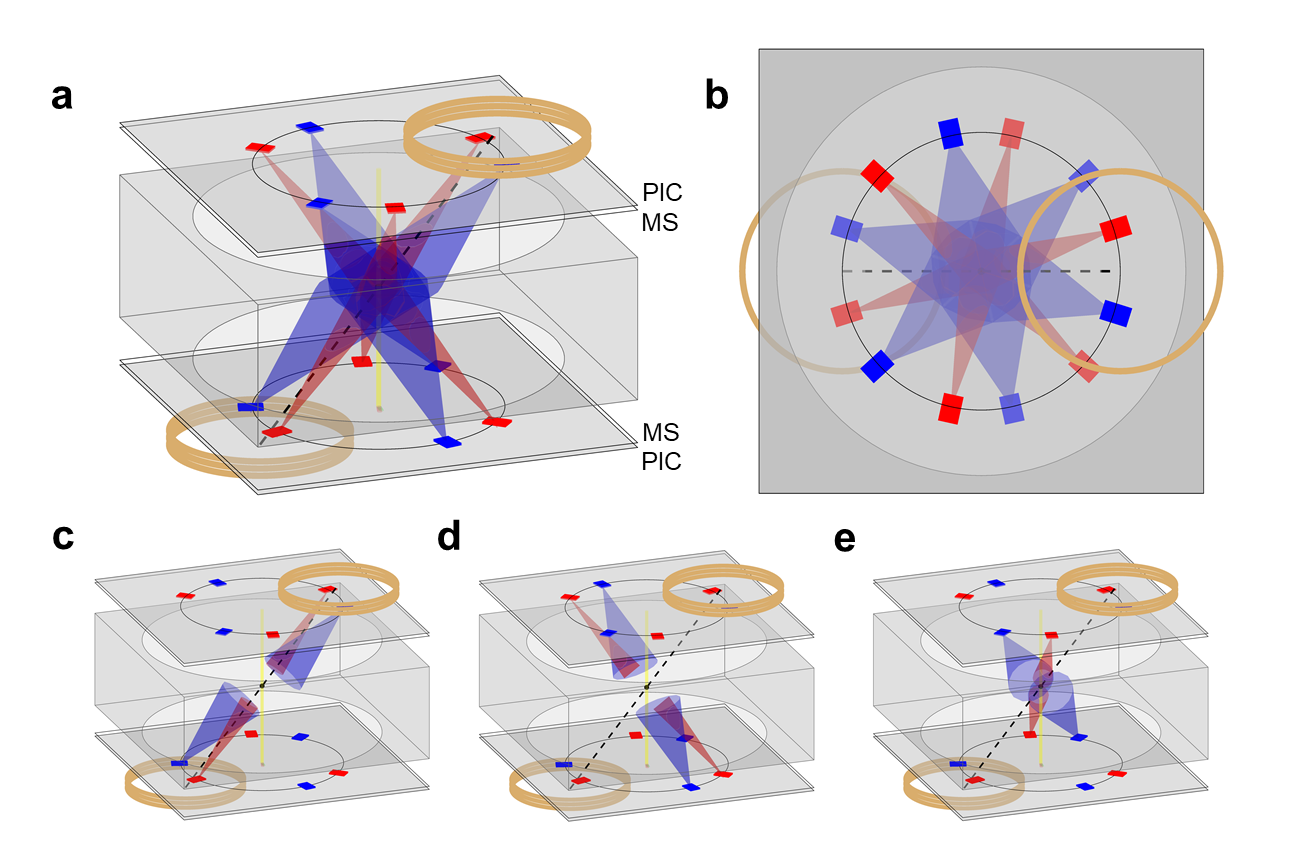
**

**Figure S2. Double-sided PIC design**. **a** Perspective view of the blue and red MOT beams along with the lattice and clock beams (yellow). The black line denotes the orientation of the magnetic field coils, which is aligned to the midpoint of the left-hand circularly polarized beam pairs. **b** Top perspective view of the design. **c-e** Perspective view of the design with different beam pairs shown.


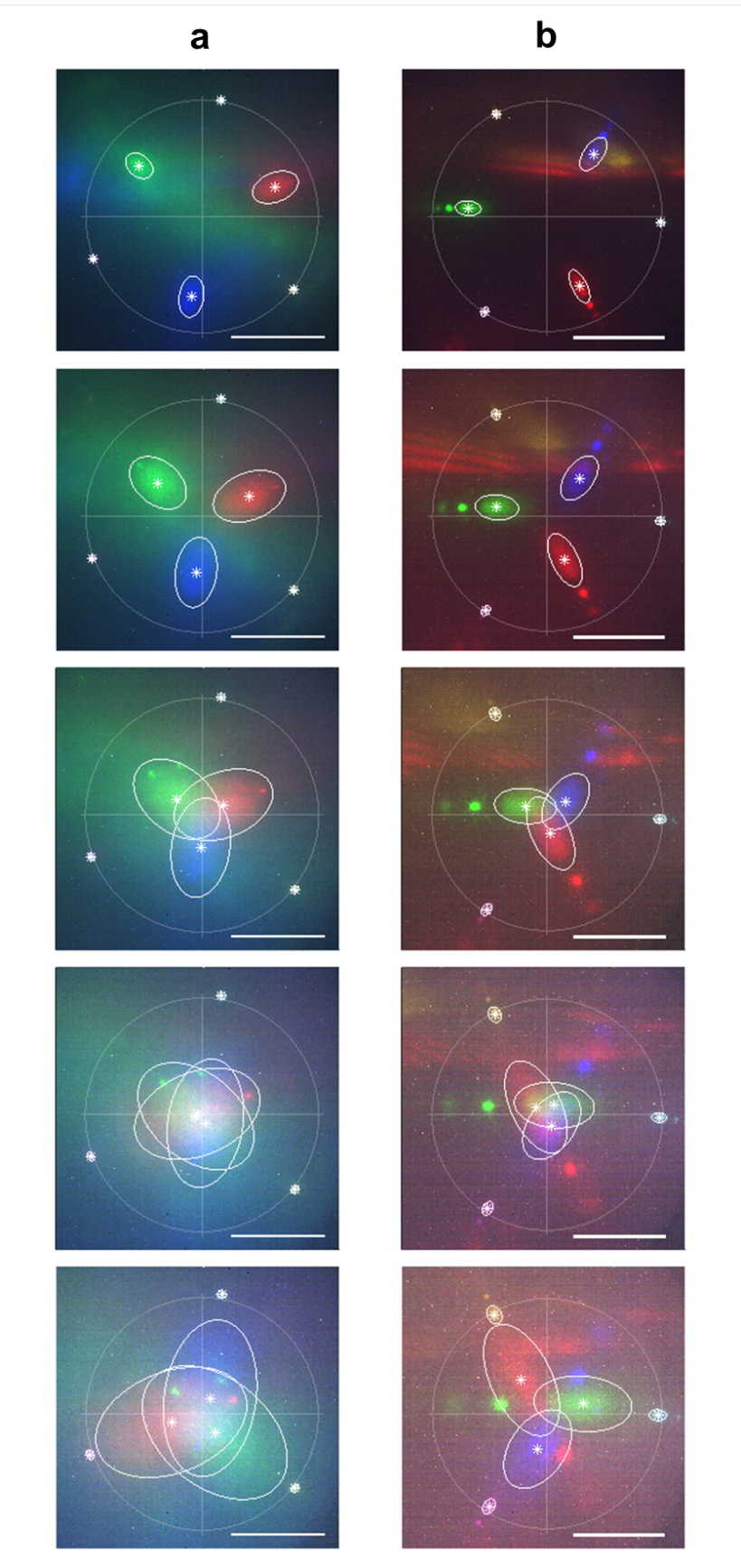


**Figure S3. MOT beam overlap measurements**. False-colored images of all 12 MOT beams with tracked positions and fitted elliptical beam waists at increasing heights. Heights are 3.75 mm, 6.25 mm, 8.75 mm, 11.25 mm, and 13.75 mm above the chip surface (top to bottom). Scale bar is 1 cm. **a** Blue MOT beams. **b** Red MOT beams, which show signs of azimuthal misalignment. Figure panels combine images taken of each beam separately, which are separately rescaled in intensity to the maximum intensity of the tracked beam. Higher order beams appear saturated.


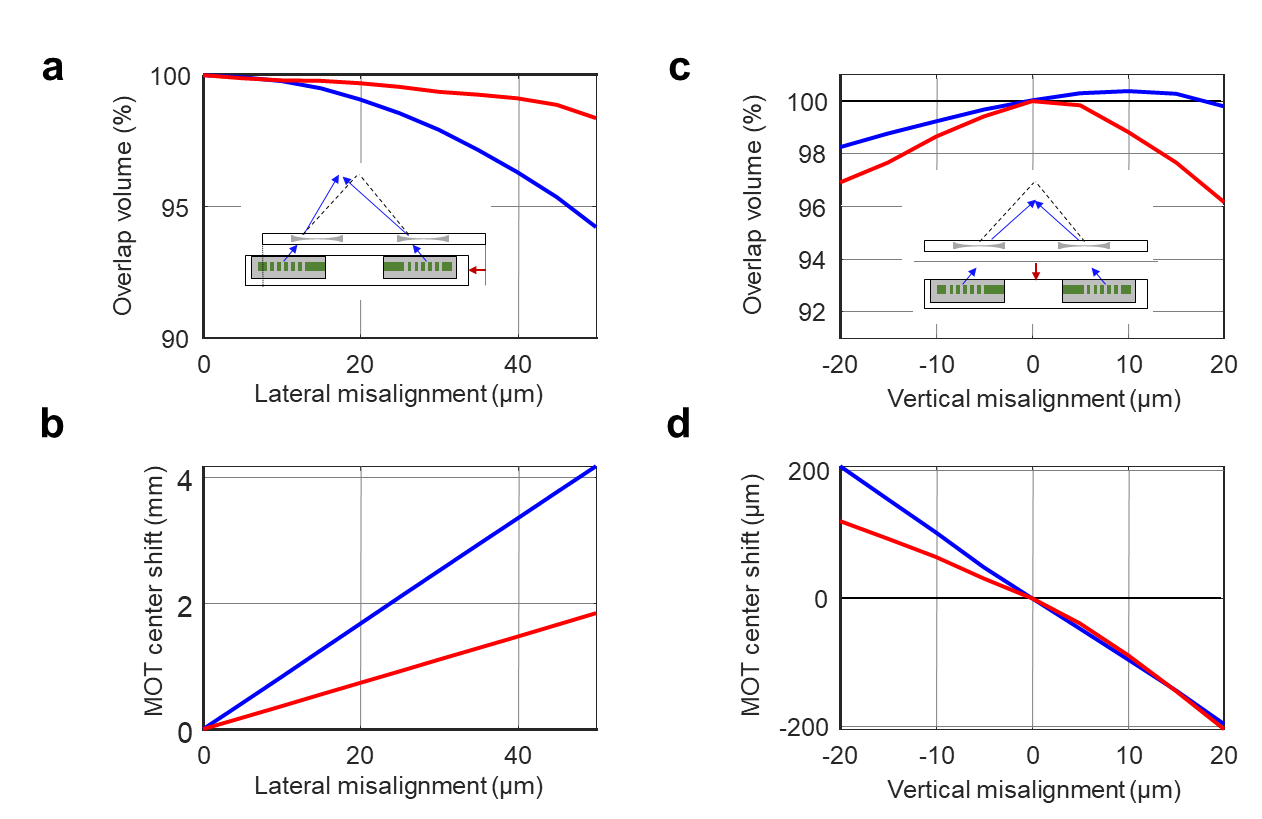


**Figure S4. MS and PIC chip misalignment sensitivities**. **a** Normalized overlap volume for the 461 nm (blue) and 689 nm (red) MOT as a function of lateral MS and PIC misalignment. Inset depicts the lateral misalignment. **b** Lateral shift of the MOT center with lateral MS and PIC misalignment. Experimental lateral misalignments are typically less than 10 µm. **c** Normalized overlap volume as a function of vertical MS and PIC misalignment. Inset depicts the vertical misalignment. **d** Vertical shift of the MOT center with vertical MS and PIC misalignment. Experimental vertical misalignments are much less than 1 µm, due to the precision of spin coating used to create the 9 µm thick SU8 adhesion layer. The MOT geometry is much less sensitive to vertical misalignments than lateral misalignments.


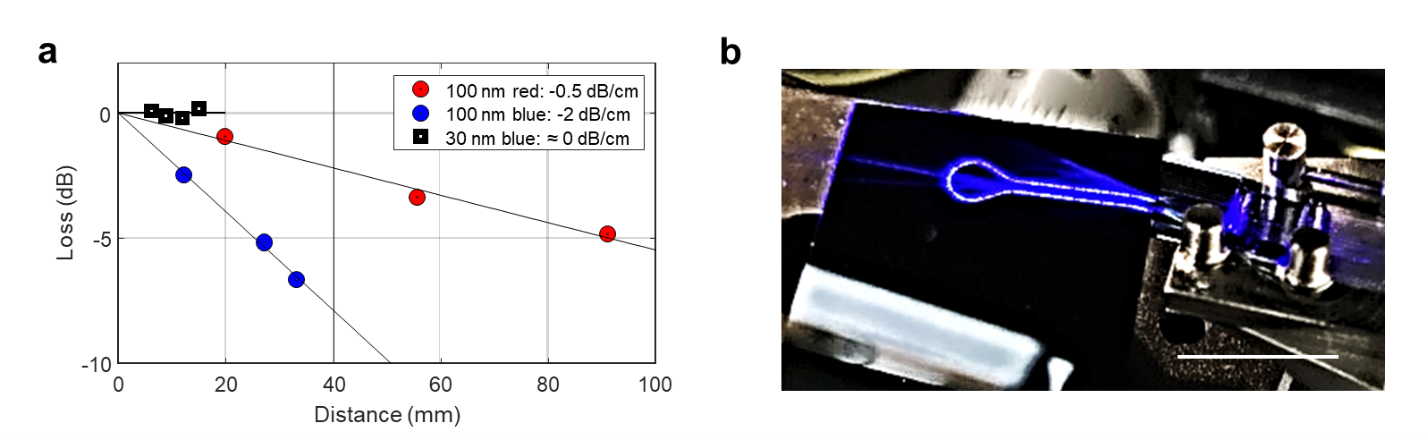


**Figure S5. Waveguide propagation losses. a** Measured propagation losses at 698 nm (red) and 461 nm (blue) for a MOT chip device compared to propagation losses at 461 nm for a 30 nm nitride test chip (black). **b** Image of a 30 nm nitride test chip consisting of a loopback design illustrating low propagation losses. Scale bar is 1 cm.
